# The Centriole’s Role in Miscarriages

**DOI:** 10.3389/fcell.2022.864692

**Published:** 2022-03-01

**Authors:** Tomer Avidor-Reiss, Luke Achinger, Rustem Uzbekov

**Affiliations:** ^1^ Department of Biological Sciences, University of Toledo, Toledo, OH, United States; ^2^ Department of Urology, College of Medicine and Life Sciences, University of Toledo, Toledo, OH, United States; ^3^ Faculté de Médecine, Université de Tours, Tours, France; ^4^ Faculty of Bioengineering and Bioinformatics, Moscow State University, Moscow, Russia

**Keywords:** centriole, centrosome, sperm, miscarriage, fertility

## Abstract

Centrioles are subcellular organelles essential for normal cell function and development; they form the cell’s centrosome (a major cytoplasmic microtubule organization center) and cilium (a sensory and motile hair-like cellular extension). Centrioles with evolutionarily conserved characteristics are found in most animal cell types but are absent in egg cells and exhibit unexpectedly high structural, compositional, and functional diversity in sperm cells. As a result, the centriole’s precise role in fertility and early embryo development is unclear. The centrioles are found in the spermatozoan neck, a strategic location connecting two central functional units: the tail, which propels the sperm to the egg and the head, which holds the paternal genetic material. The spermatozoan neck is an ideal site for evolutionary innovation as it can control tail movement pre-fertilization and the male pronucleus’ behavior post-fertilization. We propose that human, bovine, and most other mammals–which exhibit ancestral centriole-dependent reproduction and two spermatozoan centrioles, where one canonical centriole is maintained, and one atypical centriole is formed–adapted extensive species-specific centriolar features. As a result, these centrioles have a high post-fertilization malfunction rate, resulting in aneuploidy, and miscarriages. In contrast, house mice evolved centriole-independent reproduction, losing the spermatozoan centrioles and overcoming a mechanism that causes miscarriages.

## Main Text

### Introduction

Miscarriage is a complex disease involving multiple factors in the sperm, egg, embryo, and uterus (Carbonnel et al., 2021; Klimczak et al., 2021; Thomas et al., 2021; Brandt et al., 2022; So et al., 2022). Here, we focus on the sperm centriole, a factor that has reemerged in the field of reproductive biology as a critical factor post-fertilization. Centrioles were discovered by studying reproduction in the late 19^th^ century; still, the centriole’s precise role in reproduction has proven to be a mystery (Scheer, 2014; Avidor-Reiss et al., 2020). It became evident early on that many dividing cell types require exactly two centrioles per cell, and having less or more results in abnormal or dead daughter cells (Delattre and Gönczy, 2004). The centriole’s classical role is nucleating the centrosome, which acts as the dominant microtubule organization center and was characterized during the 20^th^ century (Azimzadeh et al., 2004). However, their role remained enigmatic, as centrioles were absent in many eukaryotic life forms, such as higher plants (Wheatley, 1982; Marshall, 2009; Carvalho-Santos et al., 2010; Hodges et al., 2010). Additionally, centrosome-like structures that organize cytoplasmic microtubules can form in the absence of centrioles, as seen in yeast spindle pole bodies (SPBs), and amoebozoan nucleus-associated bodies (NABs) (Gräf, 2018). Not until the turn of the 20^th^ century did it become evident that most of our body’s cells have an essential primary cilium (Rosenbaum and Witman, 2002). Cilium nucleation represents the ancestral role of centrioles, which requires the centriole to have its distinctive design, as it serves a structural template for the cilium axoneme (Avidor-Reiss, 2010; Avidor-Reiss et al., 2012). Only at the beginning of the 21^st^ century were the molecular mechanisms of centriole duplication and their composition uncovered (Guichard et al., 2018; Winey and O'Toole, 2014), meaning that centrioles were the last classical sub-cellular structures with an unknown molecular assembly mechanism. We are now beginning to develop a detailed understanding of the function of the centriole’s individual components, though the precise role of centrioles in sperm, and early embryo development remains perplexing.

In this perspective, we will provide insight into the elusive reproductive functions of centrioles and offer a potential explanation as to why mice evolved centriole-independent reproduction. It is important to note that many other mechanisms function in the zygote and play a critical role in assuring normal embryonic development (Bury et al., 2016; Masset et al., 2021; Mitchell, 2022). Furthermore, in addition to the centrioles, there are many other differences between mice and humans during early embryogenesis, including the timing of embryonic genome activation (Niakan et al., 2012), and the role of the master regulator of cell pluripotency (Daigneault et al., 2018).

### Dividing Cells Have Precisely Two Distinct Centrioles With Evolutionarily Conserved Design

Centrioles are evolutionarily conserved organelles with characteristic structure, composition, and function in animal cells (Marshall, 2009; Carvalho-Santos et al., 2010; Hodges et al., 2010). A cell typically has two distinct centrioles: a younger and an older centriole. The younger centriole is formed in the immediately preceding cell cycle and the older centriole during past cycles (Avidor-Reiss and Gopalakrishnan, 2013; Sullenberger et al., 2020). This difference in age is coupled with distinctly different compositions and functions between the two centrioles. The older centriole is structurally mature and is therefore known as the mother centriole. It extends to form the axoneme, producing the cell’s cilium in G1 or G0 of the cell cycle. The mother centriole recruits pericentriolar proteins to form the centrosome in preparation for cell division during the G2 and M phases of the cell cycle. The younger centriole is structurally immature and is referred to as the daughter centriole. Though it is linked to the mother centriole, its role in G1 of the cell cycle is unclear. However, once a cell commits to cell division, like the mother centriole, the daughter centriole duplicates in S phase, and forms a procentriole. After the subsequent M phase, the daughter centriole matures to become the mother centriole of one of the daughter cells generated during cell division. Therefore, to maintain this cycle of centriole duplication, dividing cells require two centrioles during normal animal development and physiology, and centriole formation defects result in embryo death or abnormal offspring.

### Sperm Have Two Centrioles, but the Egg Does Not

Like other cell types, early mammalian egg cells (pre-pubertal oocytes) have two centrioles, but they degenerate, and disappear during egg maturation (Simerly et al., 2018). Ultimately, meiotic cell division is mediated without recognizable centrioles (Manandhar et al., 2005; Simerly et al., 2018). The fate of the centrioles is different in sperm.

Early sperm cells (spermatids) also have two centrioles, named based on their location. The proximal centriole (PC) is located near the sperm nucleus and forms a transient, cytoplasmic, axoneme-like extension known as the centriolar adjunct (Garanina et al., 2019). The second centriole, the distal centriole (DC), is located farther away from the nucleus, and forms the sperm tail axoneme. In this centriole pair, the PC is likely the mother centriole, while the DC is the daughter centriole (Grier, 1973; Alieva et al., 2018).

For a long time, it was thought that human sperm provided one functional centriole (the PC), one degraded or remnant centriole (the DC), and no pericentriolar material (PCM) to the egg (Manandhar et al., 2005; Luo et al., 2013). However, recently it became evident that human, bovine, and probably most other mammalian sperm have two functional centrioles and PCM, though they may exhibit atypical designs (Fishman et al., 2018; Amargant et al., 2021; Khanal et al., 2021; Leung et al., 2021). The PC displays slightly modified structure and composition, while the DC displays highly modified structure and composition. The usually amorphous PCM becomes the highly structured striated columns (SCs) and capitulum in the spermatozoon. The PC and DC (possibly with the SCs) form the zygotic centrosomes.

### The Two Sperm Centrioles Form Two Essential Zygotic Centrosomes in Humans and Most Animals

For a long time, it was unclear how the mammalian zygote acquired its first two centrosomes, as it was thought that sperm provided only the PC (Schatten and Sun, 2009; Avidor-Reiss et al., 2015). However, it recently became evident that spermatozoa have a second, atypical centriole, the DC. Shortly after fertilization, the two spermatozoan centrioles stay together at the base of the decondensing male pronucleus, recruiting egg PCM proteins, and forming the first zygotic centrosome, which emanates a large aster (aka, the sperm aster). Later, the PC and DC (which is still attached to the axoneme) separate to form two independent centrosomes that are first located at the junction of the male and female pronuclei and, later, at the pole periphery of the male and female parallel spindles (Fishman et al., 2018; Cavazza et al., 2021; Kai et al., 2021; Schneider et al., 2021). The two sperm centrioles appear to be essential post-fertilization, as it is impossible to achieve live birth by fertilizing the egg with only sperm heads in humans and most other mammal species (with the notorious exception of mice and other murine species–see below) (Moomjy et al., 1999; Avidor-Reiss et al., 2019). Spermatozoan and embryonic centrioles are essential in most studied invertebrates (nematodes and fruit flies) and base vertebrates (zebrafish), suggesting that centriole-dependent reproduction is the ancestral case (Avidor-Reiss, 2018; Blachon et al., 2014; Yabe et al., 2007; O'Connell et al., 2001).

### Mice Have No Centrioles in Mature Sperm or the Early Embryo

The fate of the spermatozoan centrioles during sperm differentiation (spermiogenesis in the testes) and maturation (in the epididymis) is species-specific. Unlike most other mammals, in the house mouse (*Mus musculus*), rat (*Rattus norvegicus*), and Mongolian gerbil (*Meriones unguiculatus*), members of the Murinae family, the two mature spermatozoan centrioles are undetected (Woolley and Fawcett, 1973; Chakraborty, 1979; Manandhar et al., 1998; Simerly et al., 2016; Avidor-Reiss and Fishman, 2019). Furthermore, they, or whatever remains of them, are dispensable for early embryo development, as healthy mice and phylogenetically close species (e.g., hamsters) can be born following the injection of a tailless sperm head or nucleus into the egg (Kuretake et al., 1996; Yamauchi et al., 2002; Yan et al., 2008). Also, no centriole is detected in the embryo up to the blastula stage in natural mouse reproduction (Gueth-Hallonet et al., 1993; Coelho et al., 2013; Bangs et al., 2015). Instead of round, centriole-nucleated centrosomes, after fertilization, many small acentriolar microtubule organization centers appear in the cytoplasm, and they organize the two, unique, parallel maternal and paternal spindles (Reichmann et al., 2018). This even occurs following polyspermy (multiple paternal contribution) or parthenogenesis (no paternal contribution) (Schatten et al., 1991; Courtois et al., 2012; Zenker et al., 2017). This shows that mice exhibit novel, centriole-independent reproduction.

### The Essential Role of the Sperm Centriole is Controversial

The essential role of the sperm centriole in humans and other mammals is controversial for three main reasons.

First, a common argument against the essential role of the sperm centriole in humans is that they are dispensable in mice. However, the loss of a critical biological feature in one species does not necessarily indicate the lack of significance in other animals. While the previous statement may be trivial, it is a critical point to make. To elaborate, snakes do not have legs, but legs are essential in other animals. Snakes have lost their legs through the evolution of an alternative mode of locomotion that does not rely on legs (Jayne, 2020). Yet, humans and other land animals retained the need for legs as a means of transportation (though humans evolved walking on two legs, while most other mammals maintained the ancestral mode of walking on four legs). This leg/centriole analogy raises questions as to how and why mice have evolved centriole-independent reproduction and as to what new mechanism is substituted for the performance of centriolar function. It is important to note that “mice are not humans,” and they can differ in very basic subcellular processes (Fischer, 2021).

The second reason for the controversy is that it has not been demonstrated conclusively that sperm centrioles are essential post-fertilization by eliminating their function without causing significant defects in the sperm or zygote. To accomplish this, we need to eliminate the sperm centrioles without affecting other spermatozoan structures in a model system that has sperm centrioles (i.e., not in mice or rats); this requires experimentation on non-traditional model systems. Alternatively, this could be accomplished if efficient *in vitro* spermatogenesis could be achieved in humans or other models with sperm centrioles.

Finally, in the absence of sperm centrioles, an activated egg can be stimulated to form a parthenote (an embryo developed from an unfertilized egg) (Balakier and Casper, 1991). However, the mammalian parthenote does not reach live birth and usually dies by the blastocyst stage (Wininger, 2004; Paffoni et al., 2007; Amargant et al., 2021). Parthenotes that undergo division eventually gain centrioles through the process of *de novo* centriole synthesis. However, this process is highly erroneous, and parthenogenic cells (which do not inherit sperm centrioles) often have abnormal centriole numbers (Brevini et al., 2012). The presence of too many or too few centrioles can lead to chromosomal instability, cell death, and, ultimately, embryo developmental arrest (Bhak et al., 2006; Sir et al., 2013; Godinho et al., 2014). Additional comparative studies of both non-murine and mouse parthenogenic centrioles are necessary to determine the stage at which centrioles form, the number formed, and their function.

Research into this controversy has revealed at least two mechanisms that can organize the microtubule cytoskeleton in humans and most other mammals. One is a dominant mechanism mediated by the centriole/centrosome. The dominance of this mechanism is apparent in polyspermic zygotes, which are forced into multipolar spindle assembly due to the presence of a higher-than-normal number of centrosomes (Kai et al., 2021). The second is a complementary/compensatory mechanism mediated by the chromosomes and a set of regulatory and motor proteins. This process is beyond the scope of this manuscript, and more information about it can be found in (Mogessie et al., 2018; Geisterfer et al., 2021; Kiyomitsu and Boerner, 2021). Therefore, it is possible that non-murine zygotes, unlike mouse zygotes, require two centrioles to produce a viable embryo because they are more dependent on centriole-based mechanisms.

### Human and Bovine Have a Naturally High Miscarriage Rate

**FIGURE 1:**
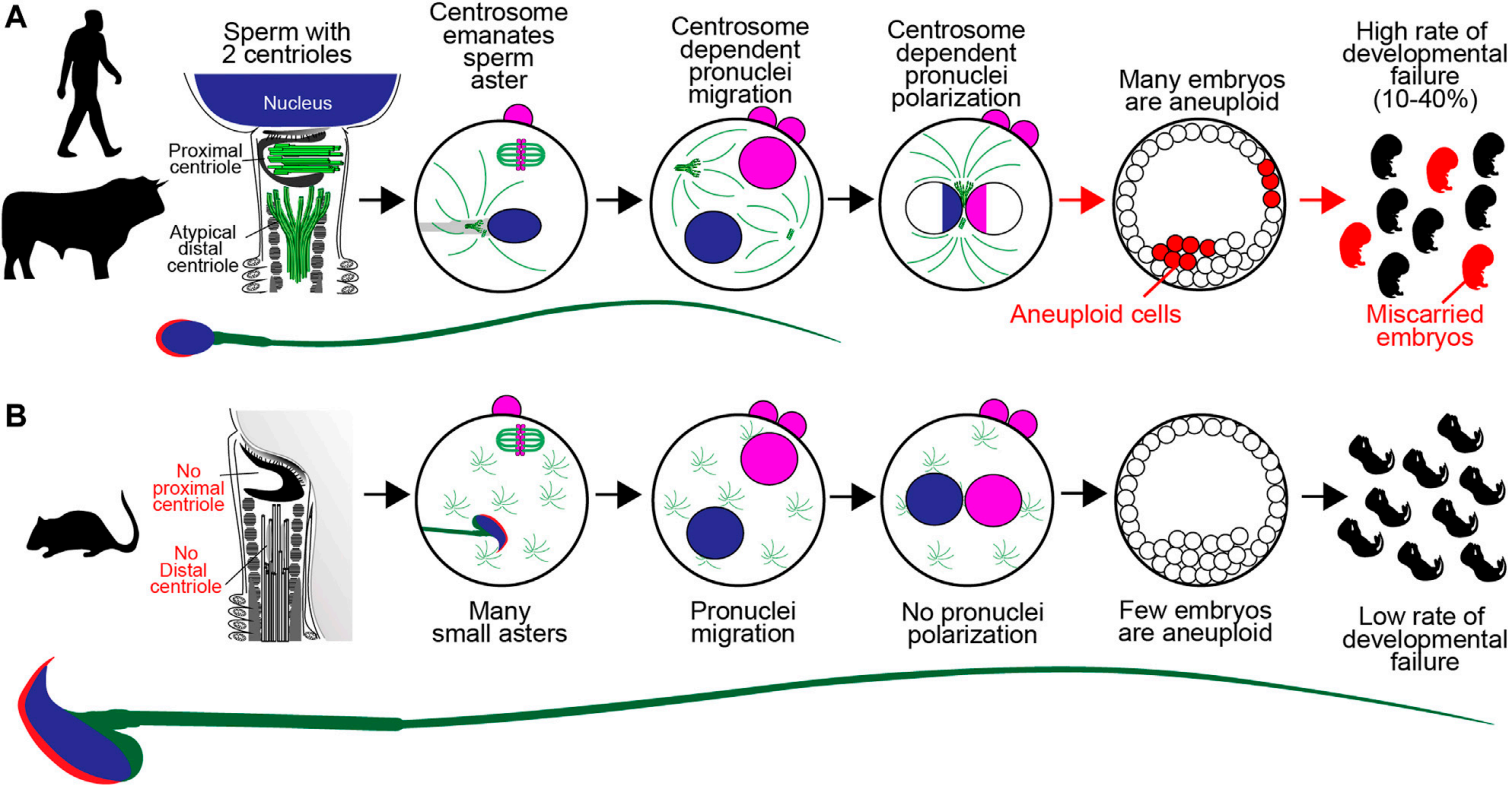
A Model of Sperm Centriole Function Post-fertilization. **(A)** In human, bovine, and many other mammalian species, the sperm has two centrioles: the proximal centriole and atypical distal centriole. During fertilization, spermatozoan centrioles are brought into the oocyte, where they form the large sperm aster, or the zygotic centrosome, and which helps pronuclear migration. The initial, single zygotic centrosome breaks into two centrosomes that move to the interface between the two pronuclei, where they polarize the paternal genome (blue) and maternal genome (pink). Failure of the sperm centrioles (red arrow) or zygotic centrosome results in embryo aneuploidy (red x) and embryo developmental failure, which are common in human and bovine. **(B)** In mice, the sperm has no detectable centrioles. After fertilization, the many small, acentriolar, cytoplasmic asters in the oocyte continue to function. The pronuclear genomes are homogenous. Embryo aneuploidy is very low, and embryo development is robust.

One potential explanation for the disappearance of centrioles in mouse reproduction is that their presence may promote early embryo aneuploidy and miscarriages. In human and bovine, reproduction is associated with a high rate of defective cell division in the early embryo (Figure 1A). For example, multinucleated blastomeres were present in 43–44% of human embryos at the two-cell stage (Balakier and Cadesky, 1997; Aguilar et al., 2016). Also, bovine embryos have a significant miscarriage rate of up to 48% in beef cattle (Reese et al., 2020) and pregnancy loss rates of ∼60% in dairy cattle (Santos et al., 2004). Three recent studies suggest that these early embryonic multinucleations and miscarriages may be due to zygotic centrosome dysfunction.

Kai et al., (2021), using live imaging of human tri-pronuclear zygotes, found that the first mitotic spindle formation is led by sperm centrosome-dependent microtubule-organizing centers and that sperm centrioles may cause a high incidence of zygotic division errors (Kai et al., 2021).

Cavazza et al., (2021) found that two zygotic centrosomes reside at the junction of the male and female pronuclei and cluster the parental genomes in human and bovine zygotes (Cavazza et al., 2021). Defects in centrosome location or function lead to aneuploidy, abnormal chromosome segregation, the appearance of micronuclei, and impaired embryo development. This demonstrates that zygotic centrosome function is critical for embryonic development in human and bovine.

Amargant et al. (2021) estimated that the human sperm carries 251 centrosomal proteins within its tail to the egg and found that one of these proteins (pericentrin) remains anchored to the sperm centrioles in the zygote (Amargant et al., 2021). This study also showed that injection of human sperm tails into human oocytes followed by parthenogenetic activation results in more robust early cell divisions and improves the parthenote’s development. They found that tail-injected parthenotes maintain better control of centriole numbers in their cells. This study demonstrates that the sperm tail and likely its centrioles contribute to embryo development.

Additional similarities identified between bovine and human early embryogenesis are absent in mice (Carreiro et al., 2021). Recently, it was reported that horses, whose sperm also have two centrioles, suffer from a high rate of aneuploidy in naturally occurring pregnancies (Shilton et al., 2020).

Together, these recent studies point to a critical role of centrosome function in the embryo that is often jeopardized, resulting in a reduction in reproductive efficiency.

### Unlike Human and Bovine, Mice Have a Naturally Low Miscarriage Rate

House mice have a very low rate of mosaic aneuploidy in preimplantation embryos (about 25% of embryos, compared to 70% in humans) (Lightfoot et al., 2006), which plays a role in their negligible rate of naturally conceived miscarriages of less than 1% (Li et al., 2011; Yang et al., 2019; Sandalinas et al., 2001; Rubio et al., 2007). Also, in contrast to the much higher rate observed in humans, the spontaneous occurrence of aneuploidy in mice is rare, affecting less than 1% of embryos (Lightfoot et al., 2006; Bond et al., 1983). For example, resorbed embryos were rarely observed in wild-type females at 11.5 days postcoitus (Figure 1B), and 0 of 90 one-cell zygotes displayed chromosomal aberrations (Yuan et al., 2002). Only 8% of mouse IVF embryos (n = 36) failed to develop into morphologically normal blastocysts, and only 9.7% of blastomeres had significant chromosome segregation errors, such as lagging chromosomes (n = 72) (Bolton et al., 2016). Therefore, it is possible that mice evolved centriole-independent reproduction to improve early embryonic development efficiency. Centrioles appear in mice just before the embryo needs to form a cilium to support essential developmental processes, such as determination of the embryo’s left-right axis. It is important to note that the house mice used in laboratories are highly inbred, multiparous mammals, compared to human and some bovine, which are outbred mammals that typically give birth to single offspring. Therefore, it would be important to study miscarriages in wild mice populations and other multiparous species with paternal sperm centriolar inheritance, such as rabbits (Vandenbergh, 2000).

### Atypical Sperm Centrioles Evolved Independently in Many Animal Groups, Suggesting Convergent Evolution

Centrioles with atypical structures evolved independently in multiple animal clades, including insects, mammals, and certain fish.

In insects, the DC varies from having 20–50 singlet tubules in the fungus gnat *Sciara coprophila* (Phillips, 1967), to having nine collapsed triplet-microtubules in *Drosophila melanogaster* (Khire et al., 2016), to having nine doublet-microtubules in *Tribolium* (Fishman et al., 2017). Discovered only recently, the PC looks alien in insects. In some insects, like *Drosophila,* the PC has no microtubules at all (Blachon et al., 2009; Gottardo et al., 2015; Khire et al., 2015; Dallai et al., 2017; Fishman et al., 2017; Uzbekov et al., 2018). Still, the *Drosophila* zygote inherits both sperm centrioles (Blachon et al., 2014). These centrioles recruit PCM, form a centrosome that emanates astral microtubules, duplicate to create new centrioles, and localize to the spindle pole (Khire et al., 2016).

In non-murine mammals, such as bovine, the PC maintains the canonical centriolar structure with some modifications (Sutovsky and Schatten, 2000; Leung et al., 2021), like the loss of canonical centriolar proteins, such as Cep295 and RTTN (Fishman et al., 2018). The PC is asymmetric, with triplet microtubules of unequal lengths (Leung et al., 2021). In these mammals, the DC is highly modified and was thought to be degraded (Manandhar et al., 2000). Recently, it was shown to be made of splayed microtubules that associate with novel rod and bar structures (Fishman et al., 2017; Khanal et al., 2021; Leung et al., 2021). Still, the two sperm centrioles are inherited by the bovine zygote, where they recruit PCM, form a centrosome that emanates astral microtubules, duplicate to form new centrioles, and localize to the junction between the male and female pronuclei and later to the vicinity of the zygote’s spindle poles.

The DC is canonical in six distantly related fish clades, but the PC is either atypical or undetected (Turner et al., 2017). While it is possible that these species lost their PC, another, more likely explanation is that the PC is modified to the point of being unrecognizable. This alternative explanation will require further investigation.

Altogether, two canonical centrioles are present in many basal animal species with primitive form (aka aquatic sperm) (BACCETTI, 1985; Jamieson, 1991), indicating that atypical centrioles evolved independently at least eight times in animals and may represent convergent evolution. However, the selective force underlying this convergence is unclear. One common feature of animal groups with atypical sperm centrioles is internal fertilization. The sperm of animals with internal fertilization show greater divergence in structure and morphology (Baccetti, 1986; Kahrl et al., 2021), indicating that atypical centriolar diversity may have coevolved with diverging sperm structure and morphology. To test this hypothesis, it would be essential to determine the specific contribution of atypical centrioles to sperm physiology.

### Sperm Centrioles Form a Dynamic Basal Complex in Bovine

As discussed above, a critical question is: why do sperm have an atypical centriole? The answer for that may vary among animal groups since each evolved independently. Revisiting our leg/centriole analogy, forelegs in basal mammals can become hands in humans or wings in bats (Krubitzer and Seelke, 2012); they were lost in the pectoral fins of dolphins (Cooper et al., 2007) and became flippers in sea lions (Reidenberg, 2007). Recently, we studied in detail the bovine atypical centriole and found that, together with other sperm neck structures, it forms a mechanical link that couples tail beating, and head kinking motions. This mechanical link, called the dynamic basal complex, (DBC) (Khanal et al., 2021) is comprised of the sperm’s specialized PCM (the striated column and capitulum), the PC, and the atypical DC.

In mammals, the transfer of force from sperm tail to head involves several neck components that work together. The striated column and DC connect to the tail independently from each other, then interact with the PC and capitulum. Finally, the PC and capitulum form a complex attached to the head. Since the insect PC is atypical (proximal centriole-like (PCL) structure), one question that arises is whether it serves the same function as it does in mammals (Gottardo et al., 2015; Khire et al., 2016). This is not likely the case in *Drosophila*, as the DC connects directly to the tail axoneme and the head, while the PCL is found in the sperm neck cytoplasm to the side of the DC, and does not appear to be part of the same mechanical linkage. However, the location of the PCL just above the mitochondria derivative that spans the tail in parallel to the axoneme may allow the PCL to connect the tail to the head in parallel to the axoneme/DC/head linkage.

Another question is: why does the sperm DC show such large differences in size between species with centriolar reproduction even though the overall sizes of their sperm are similar? For example, the bovine DC is twice as long as the human DC, based on rod protein length (Khanal et al., 2021). This situation may be analogous to some mammals having short legs and others having long legs. While leg size tends to correlate with animal size, this is not always the case. The jerboa has longer legs than other rodents of similar size so that they can move more quickly and avoid predators (Chan, 2015). One possibility is that modifications to centriolar structure allow the DBC to adapt to the unique challenges of the female reproductive tract (FRT). For example, unlike human and bovine, rabbit have induced ovulation, and the sperm reach the site of fertilization before the eggs (Fischer et al., 2012). Humans have a vagina connected to a central-chamber uterus (Suarez and Pacey, 2006), bovine have a vagina that is connected to a uterine body that splits into two uterine horns (Tung and Suarez, 2021), and rabbits have a vagina connected to two uteruses (a bicornuate duplex uterus) (Fischer et al., 2012). This diversity in reproductive anatomy may impose complex evolutionary pressures that modify sperm physiology and centriolar structure/function.

### Sperm Centriolar Defect Contributes to Infertility and Miscarriages

Since centrioles are present in mature sperm and function in the zygote, they may have a critical role in fertility, and normal embryo development. The dogma in human reproductive biology is that sperm centriolar dysfunction will result in fertilization failure (Asch et al., 1995; Simerly et al., 1995; Rawe et al., 2002). This idea arises from the early role of sperm centrioles in forming a sperm aster that facilitates male and female pronuclear congregation (Rawe et al., 2002). Sperm with severely abnormal centriolar structure fail fertilization (Chemes et al., 1987; Chemes et al., 1999; Chemes and Alvarez Sedo, 2012). Still, sometimes, polyspermic oocytes can form bipolar spindles with multiple clustered centrosomes at their poles and divide normally (Wentz et al., 1983). This idea also extends from the fact that fertilization of the egg by multiple sperm results in centriole-induced multipolar spindles and abnormal cleavage (Kai et al., 2021). However, with mild centriolar defect (i.e., centrioles are present but are partially impaired), zygote division can continue, but aneuploidy can occur, resulting in embryo death at a later stage (Cavazza et al., 2021).

Considering that the zygotic centrosome is a composite of sperm centrioles, male centrosomal proteins, and egg PCM proteins, the male and female contributions may compensate for each other’s deficiencies (Schatten, 1994; Amargant et al., 2021). Therefore, having mildly abnormal sperm centrioles can also be exacerbated by weakened eggs, resulting in failed embryonic development. This may explain why donor sperm particularly benefits older women in some cases (Bortoletto et al., 2021).

## Conclusion

The precise role of centrioles in the sperm and early embryo remains enigmatic due to challenges in its study. However, recent data suggest that the sperm provides two functional centrioles needed for normal chromosome segregation during cleavages and maintenance of normal early embryo development in human and bovine. For that, the sperm centrioles recruit maternal egg proteins and form zygotic centrosomes, which interact with paternal chromosomes. This process is sensitive, and minute errors could cause alterations in centrosome function, aneuploidy, and miscarriages. More research is needed to confirm this recent observation and understand their mechanism and role in miscarriages in human, bovine, and additional mammalian models. Mouse embryos may have found a way to develop independently of centrioles, possibly by enhancing the alternative mechanisms that exist also in the human zygote, thereby eliminating the dependency on sensitive processes and simultaneously increasing the robustness of embryonic development and fecundity. However, little is known about how this independence was achieved. More data is needed on centriole status and miscarriage rates in other species, specifically in the rodent lineage that led to house mice. More research is required to develop methodologies to overcome research challenges and test this hypothesis.

## Data Availability

The original contributions presented in the study are included in the article/Supplementary Material, further inquiries can be directed to the corresponding author.
